# Two decades of body length measurements of larval and juvenile fish populations in English rivers

**DOI:** 10.1038/s41597-024-04127-w

**Published:** 2024-11-22

**Authors:** Rachel F. Ainsworth, Lauren H. Vickers, Jonathan D. Bolland, Marie J. Taylor, Jon P. Harvey, Richard A. A. Noble, Ian G. Cowx, Andy D. Nunn

**Affiliations:** Hull International Fisheries Institute, School of Natural Sciences, University of Hull, Hull, HU6 7RX UK

**Keywords:** Freshwater ecology, Population dynamics, Conservation biology, Biodiversity

## Abstract

Long-term datasets provide context and understanding of complex ecological processes, including temporal variations in species diversity and ecosystem dynamics. This dataset is comprised of body length measurements (mm) of more than 380,000 larval or juvenile fish of 30 species from five English river catchments collected almost monthly over two decades. Such information can be used to determine growth rates, future recruitment success, population structure and compliance with monitoring protocols and conservation objectives. The dataset provides a baseline for analysing the impacts of anthropogenic disturbances such as climate change, pollution and habitat degradation, and, given that many fish populations are size structured with a positive relationship between fish body length and various biological attributes such as swimming ability, survival and fecundity, it will be invaluable for investigating natural and human- induced disturbances.

## Background & Summary

Ecological monitoring programmes can provide valuable insights for understanding and managing ecosystems, with their significance and usefulness increasing over time^1–5^, as long-term datasets provide context for understanding complex ecological processes across broad temporal scales. They provide a baseline for evaluating ecological responses to anthropogenic disturbances, such as climate change, pollution, habitat degradation and overexploitation, and can be used to inform management and evidence-based policy and decision making^2–5^. For example, long-term fish population dynamics, hydroacoustic, water chemistry and water temperature datasets from Windermere, UK, have been valuable in understanding the causes of historical changes in the fish assemblage due to exploitation^6^, species introductions, chronic pollution and habitat degradation^7,8^, and climate change^8–10^.

Monitoring of fish populations often includes measuring the body length of individuals captured. Through this method, the age structure of the populations can be determined, which can subsequently be used to determine compliance with monitoring protocols, such as the Common Standards Monitoring Guidance^11–13^ and can be used to examine growth rates and identify missing age classes^14^. Fish length data can also be used to determine overwinter survival rates and predict future recruitment success and cohort sizes^14,15^.

Larval and juvenile fish surveys were conducted in five English river catchments (Trent, Yorkshire Ouse, Ancholme, Don and Warwickshire Avon) over two decades (1999–2018). For the majority of the period, surveys were conducted on a monthly basis, making both annual and seasonal analyses of size structure, growth in body length and spawning periodicity possible^16^, but were fortnightly in one year and less frequent towards the end of the study period (2012 onwards). Length data for more than 380,000 larvae or juvenile fish of 30 species were collated, likely representing one of the most comprehensive datasets of its type. The majority of the data were collected during complementary and overlapping PhD studies of LHV, JDB, MJT and ADN, and are collated here in a single dataset. The corresponding fish abundance data are available in^17^.

This dataset can be used to elucidate temporal changes in larval and juvenile fish community or population structure. This dataset can also be used to investigate how fish communities have changed as a result of human disturbances, such as water quality, habitat modification, changes in land use and climate change.

## Methods

**Fig. 1:**
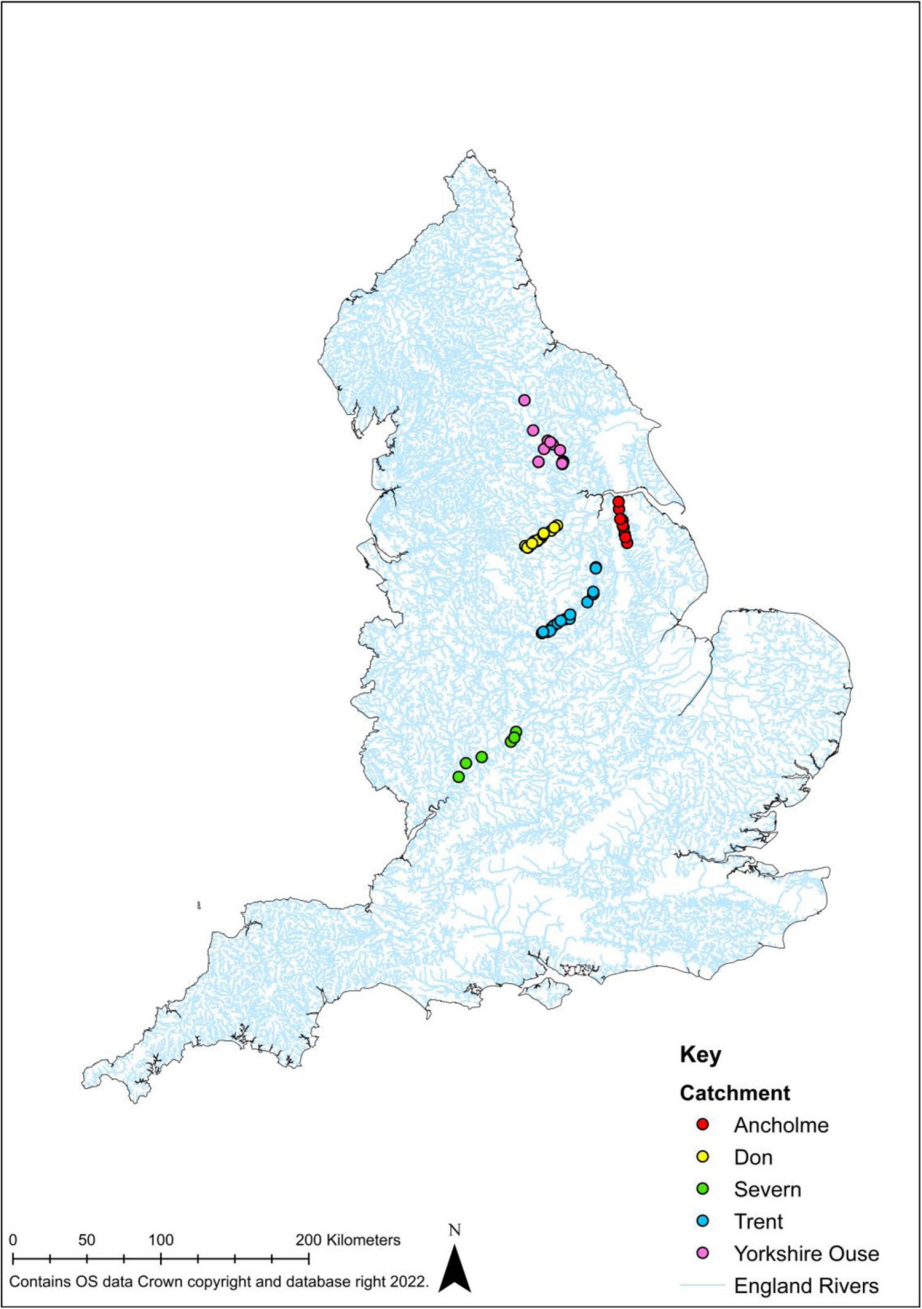
Larval and juvenile fish sampling locations in five English river catchments.

Larval and juvenile fishes were surveyed at 67 sites in five English river catchments, namely the Trent, Yorkshire Ouse (including the Swale, Ure, Nidd and Wharfe), Ancholme, Don and Warwickshire Avon (Fig. 1; Table S1). These rivers represent a range of typologies and are impacted by a contrasting array of anthropogenic pressures. The Trent, Don and Warwickshire Avon are impounded catchments with several major cities and an industrial heritage, consequently, these rivers have a history of severe pollution^18–20^ and habitat modification. In contrast, the Yorkshire Ouse drains a predominantly rural catchment, although there are a number of urban centres, impoundments and embankments, the water quality is considered good^18^. The Ancholme is an extensively modified river and has been channelised and straightened, and the flow is highly modified^21^.

**Fig. 2:**
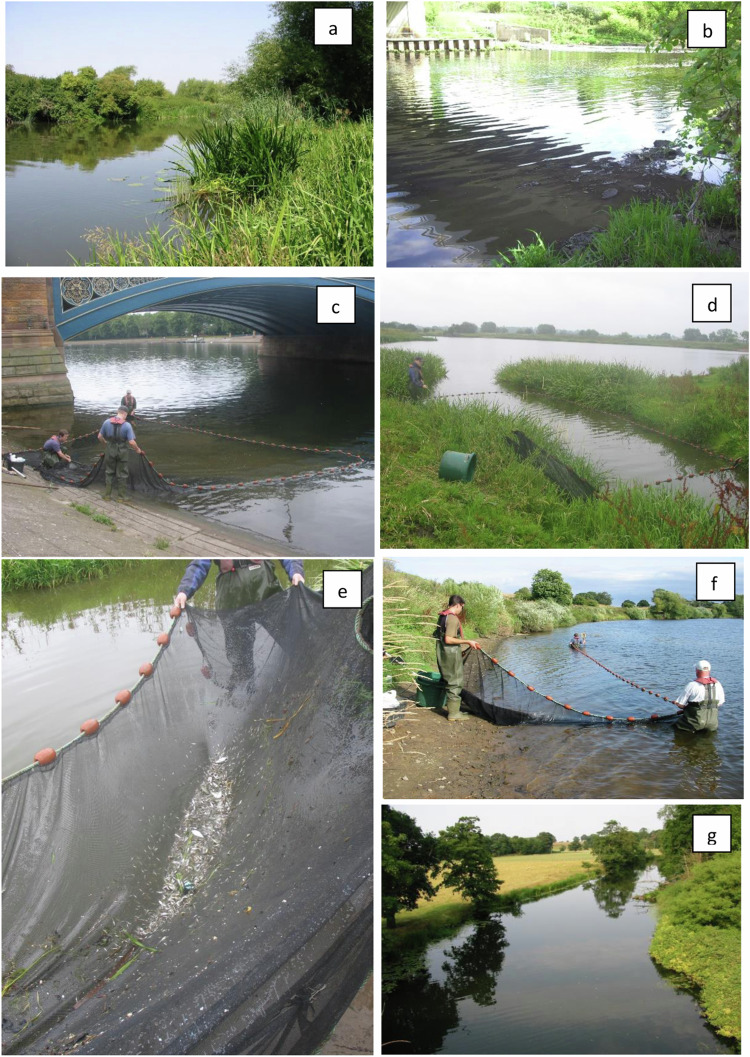
Images of sampling locations, landing sites, equipment used and fish catch. (**a**) Birlingham, (**b**) Aldwarke, (**c**) Trent Bridge, (**d**) Winthorpe, (**e**) example of typical contents of a micromesh seine net at Winthorpe, (**f**) Beningborough and (**g**) Warwick.

Surveys were conducted in the river margins, where water velocity is slowest and larval and juvenile fishes tend to aggregate^22^. Fish were captured using a 25 × 3 m micromesh (3 mm) seine net set in a rectangle parallel to the bank either by wading or from an inflatable boat (Fig. 2). This net captures fish as small as 5 mm and is the most appropriate method of catching large numbers of larval and juvenile fishes^23^, although occasionally some larger adult fish may have also been captured and measured as part of this dataset for completeness. The net was fished to the bank in the usual manner for a beach seine (Fig. 2) and captured fishes were transferred to water-filled containers prior to data collection.

Up to 100 random individuals of each fish species were measured for each survey; measuring more than 100 fish does not significantly improve the accuracy of length distributions or mean length estimates^24^. All fish were identified to species^25^, measured to standard length (mm) and released at their point of capture. The exception was the smallest larvae, which were euthanised with an overdose of methanesulphonate (MS-222) and preserved in 4% formalin solution for microscopic examination in the laboratory.

## Data Records

This dataset comprises individual body length measurements (mm) of more than 380,000 larval or juvenile fish of 30 species from five English river catchments between 1999 and 2018 inclusive. The dataset contains 384,090 rows and 13 columns. Each row corresponds to a single fish that was measured. Associated site information (site name, location, area fished (m^2^) and survey date) is reported for each row. When only a fraction of the catch was processed, the sub-sample size was reflected in the Count column (e.g. when half the sample was processed, the numbers of fish measured or only counted were multiplied by two). This enables accurate densities to be calculated as the total number of both measured and unmeasured fish is recorded. The dataset is provided in a CSV file on the Zenodo data repository^26^.

## Technical Validation

All larval and juvenile fish species were identified to species using the Freshwater Biological Association Key of Larval and Juvenile fishes^25^, which was developed using fish of known parentage in a fish farm, thereby minimising the possibility of errors from the inadvertent inclusion of hybrids. Field samples were also collected for comparative purposes and for validating the keys. This study also followed recommendations outlined in^25^ that the smallest larvae be preserved in the field and retained for microscopic evaluation for their identities to be checked and validated. Similarly, for surveys conducted in late Summer, Autumn and Winter, the fish were sufficiently large to use well established adult morphometric and meristic characteristics for identification purposes^27,28^. All fish identification was also cross checked and validated in the context of the known life cycles, phenology and habitat requirements of the various species^16,25,27,28^. Finally, at least one staff member involved with the surveys was highly experienced in larval and juvenile fish identification, and fish identified by less experienced personnel were checked and validated. During assembly of the dataset, all data entries were checked and validated by RFA and ADN, to minimise the possibility of typographical errors.

**Fig. 3:**
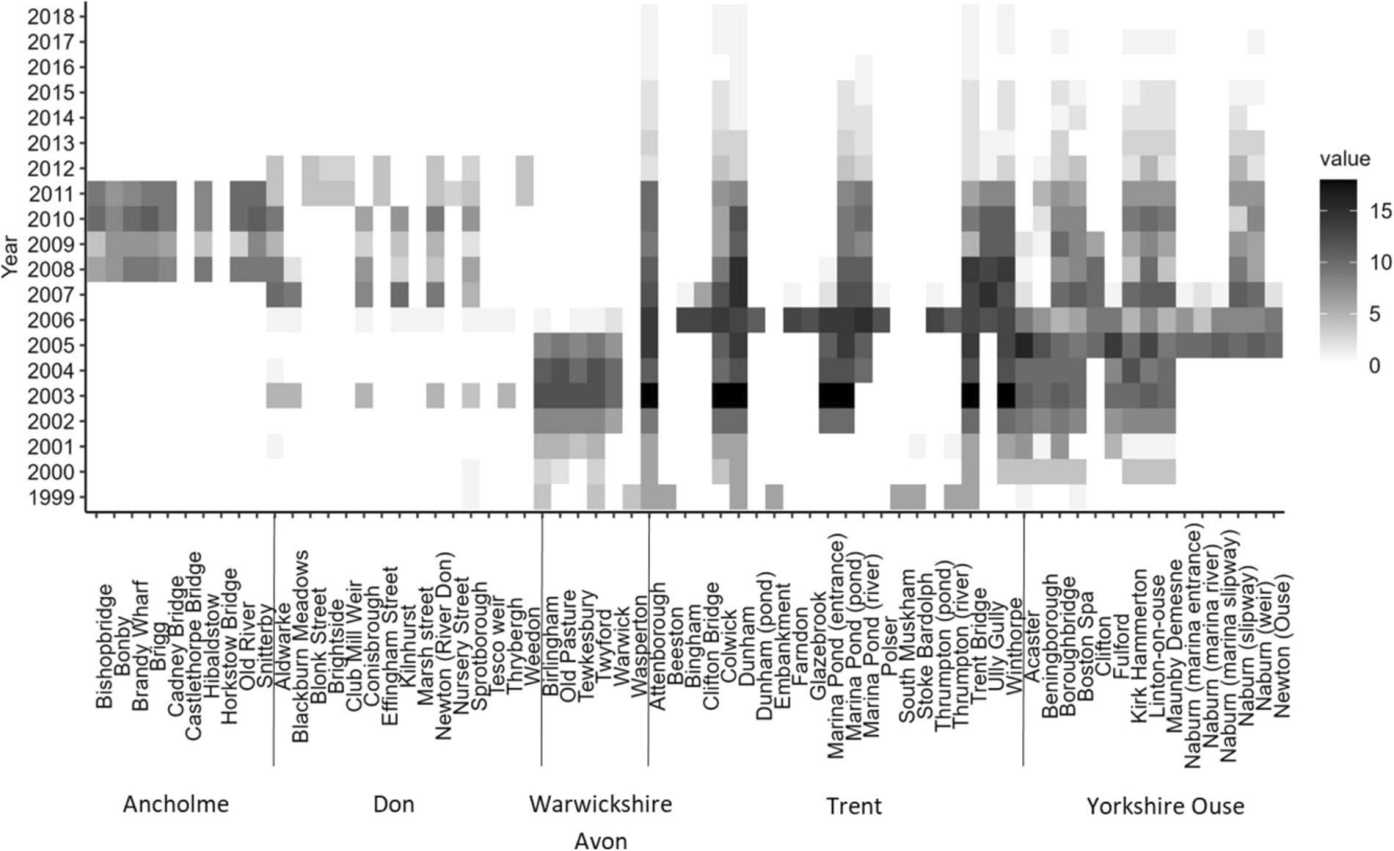
Number of surveys per site each year during the sampling period (n).

**Fig. 4:**
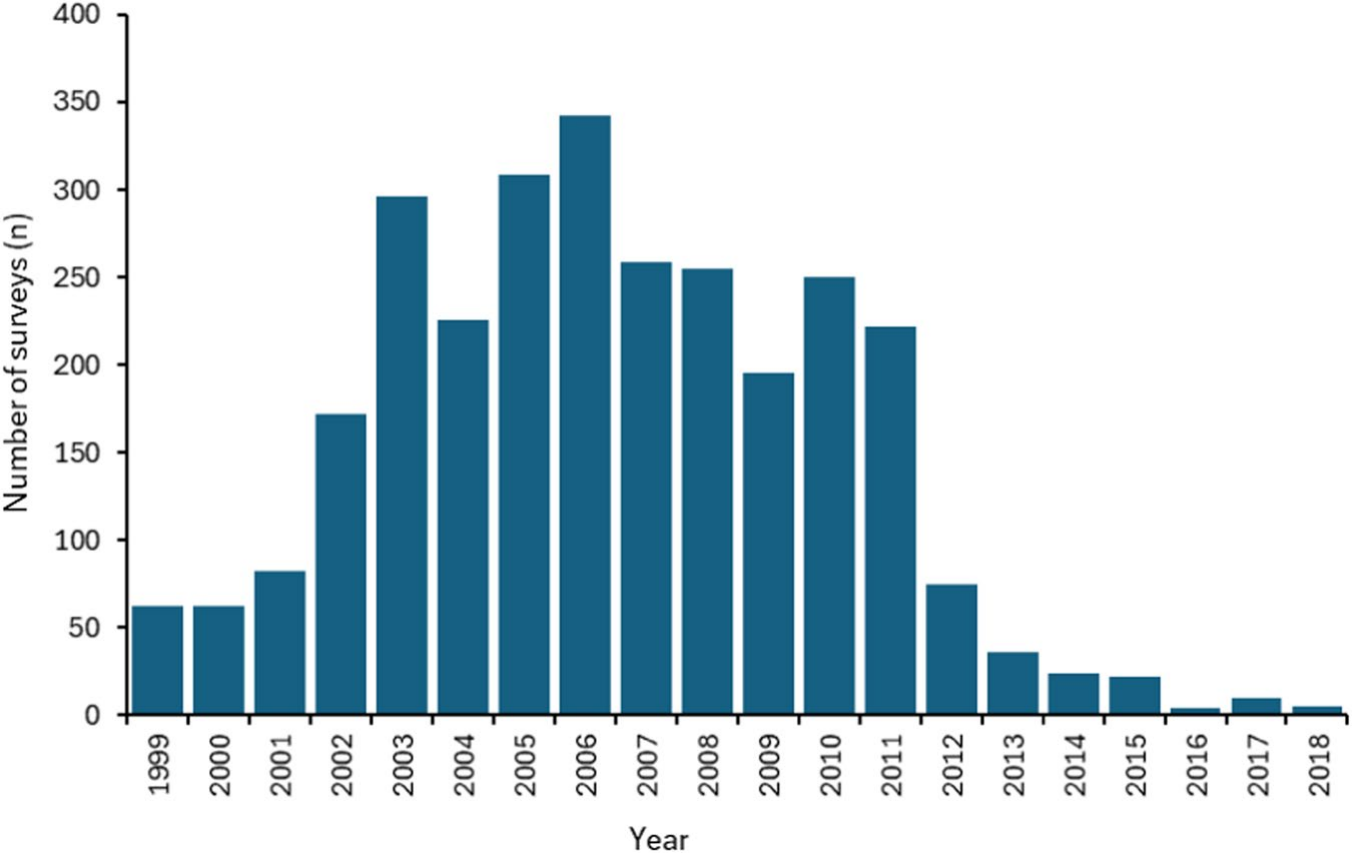
Number of surveys per year throughout the sampling period (n).

Site-specific sampling frequency varied according to the requirements of specific studies, but many sites were surveyed approximately monthly for the duration of the dataset, whereas others were surveyed only once (Fig. 3). The years 2002–2011 were surveyed the most intensively, with a mean of 254 surveys each year (minimum = 172, maximum = 341) (Fig. 4). The number of surveys at each site ranged from one to 18 per year, with Boroughbridge, Linton-on-Ouse (Yorkshire Ouse), Attenborough, Colwick, Dunham, Marina Pond (pond), Trent Bridge and Winthorpe (Trent) each surveyed on more than 100 occasions (Fig. 3). Some sites, such as Dunham, Trent Bridge and Winthorpe (Trent), were surveyed in all but one year, whereas Embankment (Trent) and Wasperton (Warwickshire Avon) were surveyed in only one year (Fig. 3). The inclusion of sites surveyed on a few occasions is warranted to enable the analysis of seasonal and or spatial variations in larval and juvenile fish assemblages.

**Fig. 5:**
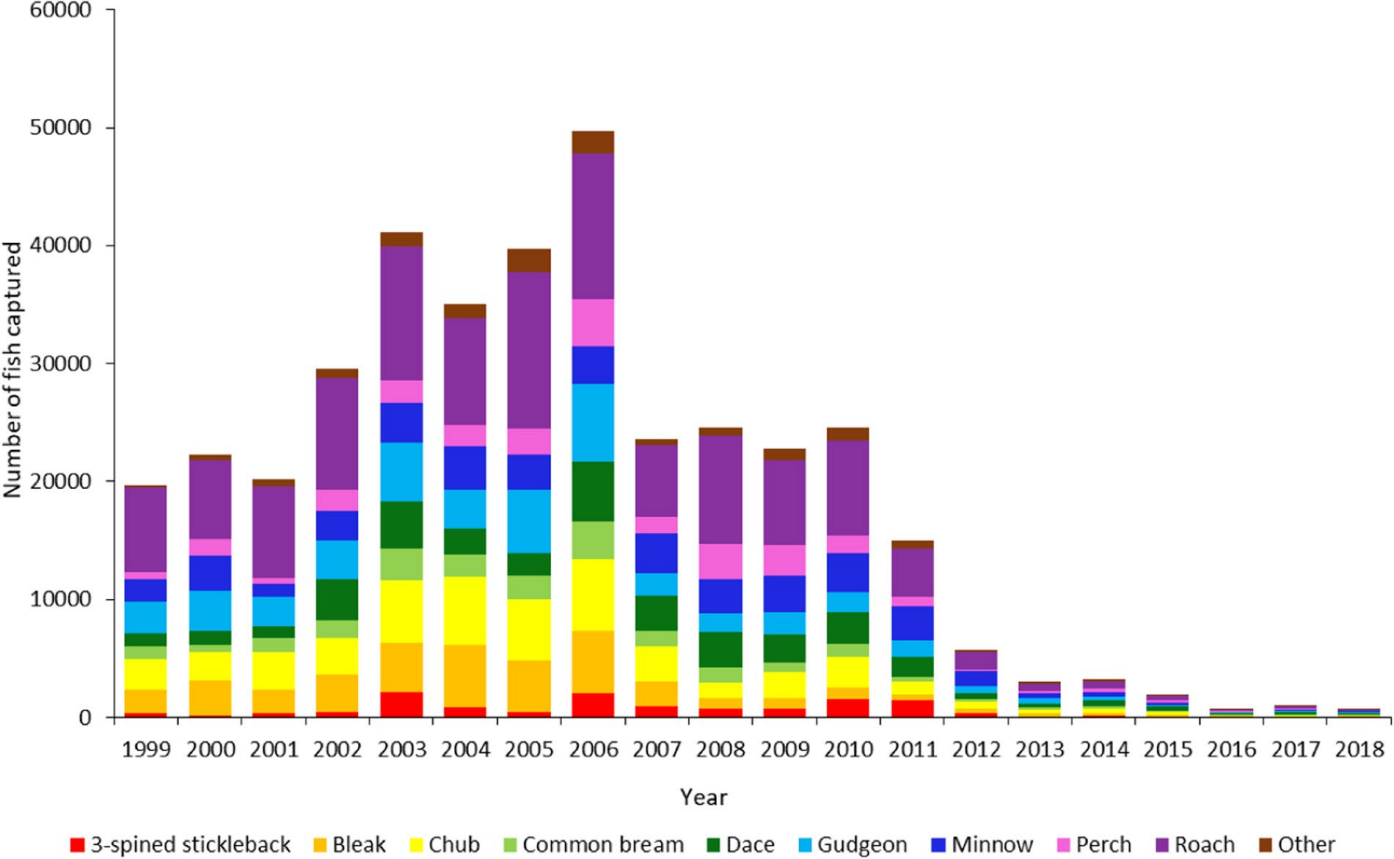
Number of three-spined stickleback, bleak, common bream, dace, chub, gudgeon, minnow, perch and roach measured by year throughout the sampling period. Other less common species aggregated into ‘Other’ category.

The number of species and individuals measured varied annually, as indicated in Fig. 5 with the number of the most common species (3-spined stickleback (*Gasterosteus aculeatus*), bleak (*Alburnus alburnus*), chub (*Squalius cephalus*), common bream (*Abramis brama*), dace (*Leuciscus leuciscus*), gudgeon (*Gobio gobio*), minnow (*Phoxinus Phoxinus*), perch (*Perca fluviatilis*) and roach (*Rutilus rutilus*)) measured per year summarised with the remaining species classed as ‘Other’. The greatest number of fish were measured between 2003 and 2006, with an annual mean ± S.D of 41,428 ± 6,120 individuals. During these years, roach was the dominant species measured, with a mean of 11,513 ± 1,761 individuals per year (Fig. 5). In total, 30 species are recorded in the dataset, the most abundant being roach, with more than 115,000 individuals measured, followed by chub, gudgeon and minnow, each with 40,000 or more measurements (Fig. 5).

**Fig. 6:**
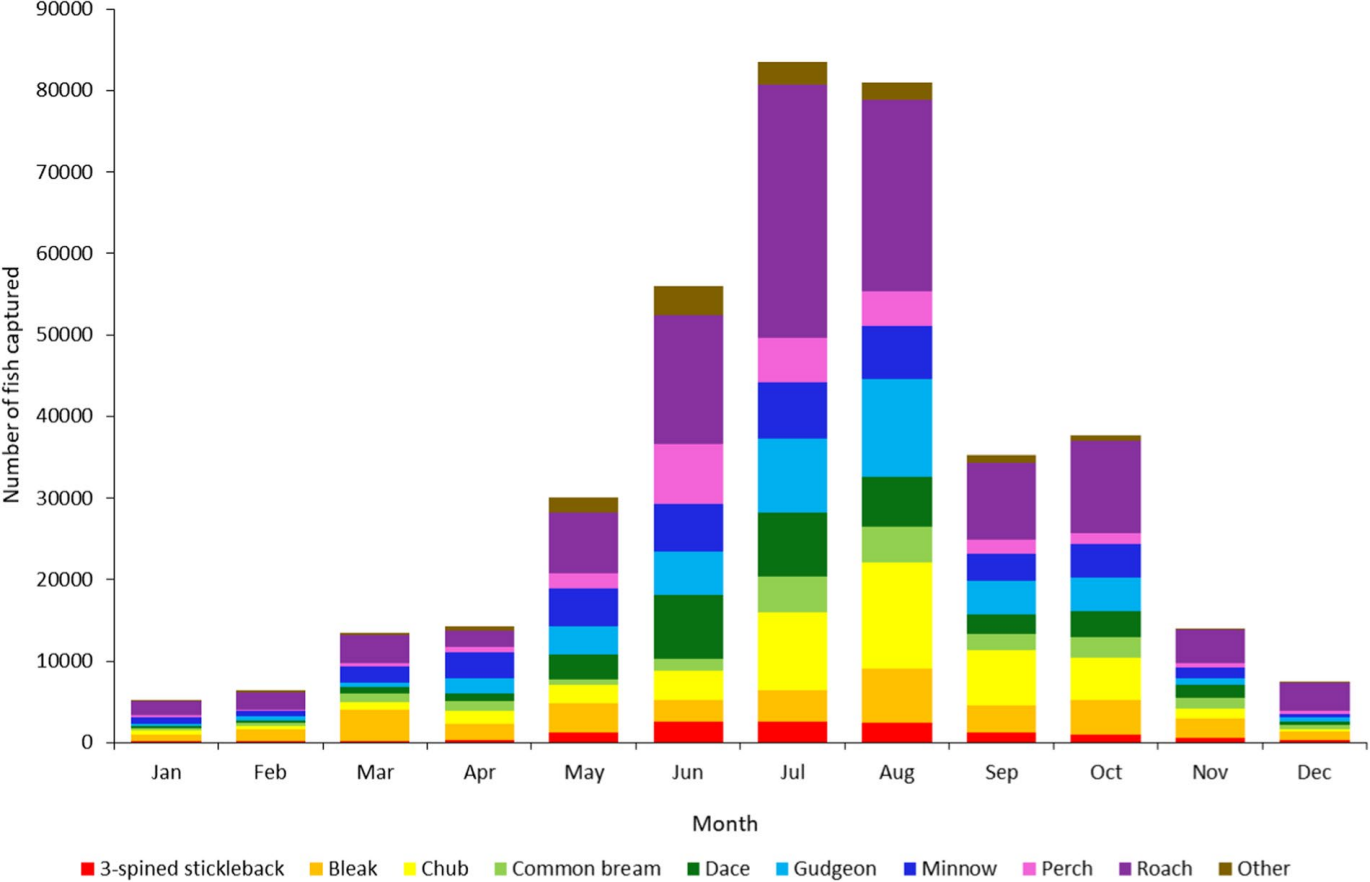
Number of three-spined stickleback, bleak, common bream, dace, chub, gudgeon, minnow, perch and roach measured per month throughout the sampling period. Other less common species aggregated into ‘Other’ category.

Seasonal or monthly variation can also be seen in the number of species and individuals measured as is indicated in Fig. 6. The mean ± S.D. number of fish measured each month was 32,029 ± 28,001 and was highest during the summer months of June-August (73,490 ± 12,410) (Fig. 6). Species such as common goby (*Pomatoschistus microps)*, common carp (*Cyprinus carpio)*, barbel (*Barbus barbus*), and pikeperch (*Sander lucioperca*) were typically only caught during the warmer months (April-October) in the UK, with all other species caught throughout the year (Fig. 6).

## Supplementary information

Table S1

## Data Availability

No custom code was used to generate or process the data for the dataset described in this manuscript. Arc GIS (ArcMap 10.8.2) was used to create Fig. 1 with site location data from Table S1. R (version 4.2.2) and R Studio (Version 2022.7.1) was used to create Figs. 3 and 4 for visual purposes only for this paper. Microsoft Excel was used to create Figs. 5 and 6.
