# Supplementary material for: Two decades of body length measurements of larval and juvenile fish populations in English rivers
Source: Sci Data. 2024 Nov 22;11:1271. doi: 10.1038/s41597-024-04127-w (PMC11584624; doi:10.1038/s41597-024-04127-w)
Supplement: Supplementary file 1 — Table S1 [file 41597_2024_4127_MOESM1_ESM.pdf]

Table of Contents

TABLE S1: SITE NAMES, LOCATIONS, AREA FISHED AND START AND END DATES (DD/MM/YYYY) OF SURVEYS FOR EACH JUVENILE SURVEY SITE. 2

Table S1: Site names, locations, area fished and start and end dates (dd/mm/yyyy) of surveys for each juvenile survey site.

Catchment	Site Name	Latitude	Longitude	Area Fished (m^{-2})	Start Date	End Date
Ancholme	Bishopbridge	53.40575	-0.45058	108	30/05/2008	30/08/2011
	Bonby	53.61256	-0.52616	80	08/09/2001	30/09/2011
	Brandy Wharf	53.45999	-0.47421	90	23/08/2001	30/09/2011
	Brigg	53.54934	-0.5027	80	30/05/2008	30/09/2011
	Cadney Bridge	53.5123	-0.49194	76	13/09/2001	30/09/2011
	Castlethorpe Bridge	53.55221	-0.51618	80	17/09/2001	17/09/2001
	Hibaldstow	53.49966	-0.48335	64	30/05/2008	30/09/2011
	Horkstow Bridge	53.65754	-0.52914	100	19/09/2001	19/09/2001
	Old River	53.54292	-0.49236	68	30/05/2008	30/09/2011
	Snitterby	53.44012	-0.46739	84	14/08/2001	30/09/2011
Don	Aldwarke	53.44534	-1.32395	75	20/12/2001	01/08/2012
	Blackburn Meadows	53.42505	-1.395	78	28/05/2003	17/04/2008
	Blonk Street	53.38582	-1.46171	120	18/05/2011	01/08/2012
	Brightside	53.4072	-1.42082	85	18/05/2011	01/08/2012
	Club Mill Weir	53.39582	-1.48714	72	18/05/2011	01/08/2012
	Conisbrough	53.48787	-1.22078	68	28/05/2003	29/10/2010
	Effingham Street	53.38849	-1.45416	101	18/05/2011	01/08/2012
	Kilnhurst	53.47216	-1.29791	78	08/08/2006	29/10/2010
	Marsh Street	53.42578	-1.36187	68	08/08/2006	08/08/2006
	Newton (Don)	53.51893	-1.16139	75	28/05/2003	01/08/2012
	Nursery Street	53.38675	-1.46621	65	30/06/2011	20/07/2011
	Sprotborough	53.50654	-1.19028	40	23/09/1999	13/07/2012
	Tesco Weir	53.43117	-1.36029	50	08/08/2006	08/08/2006
	Thrybergh	53.4632	-1.30257	45	28/005/2003	11/08/2006
	Weedon	53.41168	-1.41775	74	18/05/2011	01/08/2012
Warwickshire Avon	Birlingham	52.07539	-2.08751	70	01/07/1999	29/06/2006
	Old Pasture	52.20526	-1.64291	80	04/07/2000	20/12/2005
	Tewkesbury	51.99171	-2.15871	72	04/07/2001	29/6/2006
	Twyford	52.11227	-1.93133	80	01/07/1999	23/05/2006
	Warwick	52.26532	-1.59114	64	01/07/2002	29/06/2006
	Wasperton	52.23032	-1.60904	50	01/07/1999	15/11/1999
Trent	Attenborough	52.89553	-1.22996	84	19/05/1999	24/07/2018
	Beeston	52.91154	-1.20439	50	17/06/1999	19/10/1999
	Bingham	53.09698	-0.80521	75	07/03/2006	22/02/2007
	Clifton Bridge	52.92387	-1.16698	75	07/03/2006	08/08/2007
	Colwick	52.9476	52.9476	96	13/06/2000	24/07/2018
	Dunham	53.26116	-0.77215	96	27/05/1999	24/07/2018
	Dunham (pond)	53.25488	-0.77383	96	07/03/2006	21/08/2006
	Embankment	52.93804	-1.13695	50	19/05/1999	19/10/1999
	Farndon	53.04992	-0.86469	75	07/03/2006	22/02/2007
	Glazebrook	52.86465	-1.32558	60	07/03/2006	16/11/2006
	Marina Pond (entrance)	52.95005	-1.06081	50	15/02/2002	31/01/2008
	Marina Pond (pond)	52.95011	-1.06825	35	15/02/2002	15/10/2015
	Marina Pond (river)	52.95008	-1.06526	70	16/03/2004	02/11/2016

	Polser	52.95083	-1.0459	75	07/03/2006	22/02/2007
	South Muskham	53.09965	-0.80215	50	18/05/1999	19/10/1999
	Stoke Bardolph	52.97595	-1.03939	50	18/05/1999	30/07/2001
	Thrumpton (pond)	52.8724	-1.26752	75	07/03/2006	22/02/2007
	Thrumpton (river)	52.87854	-1.24364	50	18/05/1999	16/11/2006
	Trent Bridge	52.93893	-1.13545	80	19/05/1999	24/07/2018
	Ully Gully	52.87267	-1.31357	75	04/04/2006	07/06/2013
	Winthorpe	53.11225	-0.80329	60	13/06/2000	24/07/2018
Yorkshire Ouse	Acaster	53.89601	-1.10212	56	17/09/1999	04/08/2009
	Beningborough	53.90657	-1.34391	66	15/06/2000	30/07/2012
	Boroughbridge	54.09919	-1.39746	36	14/06/2000	17/07/2017
	Boston Spa	53.90747	-1.34543	66	25/05/1999	23/10/2015
	Clifton	53.97705	-1.12173	80	01/02/2005	24/09/2009
	Fulford	54.01263	-1.19575	75	13/06/2001	16/02/2007
	Kirk Hammerton	53.98533	-1.28625	70	14/06/2000	17/07/2017
	Linton-on-Ouse	54.03543	-1.24723	72	15/06/2000	17/07/2017
	Maunby Demesne	54.28294	-1.48387	60	14/06/2000	17/07/2017
	Naburn (marina entrance)	53.90763	-1.09274	60	01/02/2005	16/02/2017
	Naburn (marina river)	53.90943	-1.09269	75	01/02/2005	12/07/2007
	Naburn (marina slipway)	53.90761	-1.08969	75	01/02/2005	16/02/2017
	Naburn (slipway)	53.90224	-1.09285	40	01/02/2005	16/06/2015
	Naburn (weir)	53.8924	-1.09915	120	01/02/2005	17/07/2017
	Newton (Ouse)	54.02717	-1.22142	70	29/03/2005	13/06/2007
